# Evidence of *Coxiella burnetii* infection among pregnant and aborted women: A systematic review and meta-analysis

**DOI:** 10.1016/j.nmni.2025.101619

**Published:** 2025-08-05

**Authors:** Mina Latifian, Fahimeh Bagheri Amiri, Ehsan Mostafavi, Saber Esmaeili

**Affiliations:** a National Reference Laboratory for Plague, Tularemia and Q Fever, Research Centre for Emerging and Reemerging Infectious Diseases, Pasteur Institute of Iran, Akanlu, Kabudar Ahang, Hamadan, Iran; b WHO Collaborating Centre for Vector-Borne Diseases, Department of Epidemiology and Biostatics, Research Centre for Emerging and Reemerging Infectious Diseases, Pasteur Institute of Iran, Tehran, Iran

**Keywords:** *Coxiella burnetii*, Q fever, Pregnancy, Abortion, Women

## Abstract

**Background:**

Q fever infection in pregnant women and its complications have been neglected in the world and little attention has been paid to it by health systems. Therefore, this study aimed to provide evidence regarding the association between Q fever infection and its effects on human abortion.

**Methods:**

English electronic data sources were searched including PubMed and Web of Science from 1993 to December 2024; the search strategy was implemented using these keywords (in the title/abstract) were used: “*Coxiella burnetii*” OR “Q fever” and “pregnancy” OR “pregnant” OR “women” OR “miscarriage” OR “humans” OR “abortion”.

**Results:**

In this review, 30 papers of 705 candidate articles obtained from two databases, were considered for meta-analysis. The overall seroprevalence of *Coxiella burnetii* in pregnant women was 11 %, higher in low-income countries and high-risk women. Molecular detection was low (0.1 %), slightly higher in high-risk and abortion-history groups. Past infection prevalence was 62.6 %, more common in high-risk individuals.

**Conclusions:**

According to the results, the seroprevalence of Q fever among pregnant women, especially women who have a history of abortion, is significant and requires more investigations by the health providers. Active surveillance and further studies are recommended to more clearly define the epidemiology and significance of *C. burnetii* infections in pregnant women.

**Prospero registration number:**

CRD42023402863.

## 1 Introduction

Q fever is known as a worldwide zoonotic disease except in New Zealand. The infection is caused by pleomorphic obligate gram-negative bacteria called *Coxiella burnetii* [1]. Farm animals and pets are the main reservoirs of infection and infected animals shed *C*. *burnetii* to the environment through the excretion of faeces, urine, milk, and especially birth products and bacteria can persist in the soil for several months [2]. *C*. *burnetii* is a pathogen for humans, and infection transmits through the inhalation of infected aerosol. Other rare transmission routes to humans are included consumption of contaminated dairy products, direct contact with parturient of infected animals, vertical transmission, person-to-person transmission, and tick bite [3].

Q fever is generally asymptomatic. In symptomatic form of Q fever infection three clinical forms can occur including: acute Q fever, chronic Q fever, and fatigue syndrome. Acute Q fever is often self-limiting and causes flu-like symptoms, and can be associated with pneumonia and hepatitis. [2,4]. Acute Q Fever may progress to Chronic Q Fever after months or years in only 5 % of people infected. The most common complication is endocarditis. [3]. In the case of fatigue syndrome, it is said that the persistence of *C. burnetii* antigens leads to inflammation and ultimately causes serious changes in mood [5].

Clinical evidence shows that suppression of the human immune system by Human Immunodeficiency Virus (HIV), cancer, lymphoma, and pregnancy can be associated with the occurrence of chronic Q fever [6]. In pregnancy, the fetus is considered as a semi-allograft and suppression of the secondary immune system, and this condition leads to increased susceptibility to infections [7]. When the pregnant women get Q fever, the bacteria colonize the uterus, mammary glands, and placenta, and the infection can be transmitted across the placenta [8]. Trophoblast cells make up the majority of cells in the placenta, and the rest of the cells are related to immune cells, macrophages, dendritic cells, and mast cells, respectively [9]. *C*. *burnetii* can proliferate in trophoblast cells and cause an inflammatory reaction leading to placental necrosis and vasculitis, which can lead to miscarriage [10].

Q fever infection in pregnant women is associated with serious complications as well as high mortality. The first documented report of Q fever infection during pregnancy was published in France in 1990, describing five cases of pregnant women who experienced abortion or premature delivery associated with the infection [11]. Histopathological studies of placental tissue in *C. burnetii*-infected pregnancies have revealed two distinct patterns. The first is associated with symptomatic infection and is characterized by placental necrosis and a marked inflammatory infiltrate, including neutrophils and plasma cells within the decidua. The second pattern is observed in asymptomatic cases, where necrosis is absent but the placenta exhibits fibrotic changes in the chorionic villi along with evidence of haemorrhage [12].

Q fever infection in pregnant women has been associated with miscarriage, preterm birth, or low birth weight, and infrequently with fetal death, or congenital malformations. The outcome of Q fever in pregnancy depends on the trimester of pregnancy, and if a person becomes infected in the first trimester, the chances of miscarriage are greatly increased. Also, if a person becomes infected in the first trimester, the mother's risk of developing chronic Q fever increases in subsequent pregnancies. In the second and third trimesters of pregnancy, having a Q fever infection can be associated with preterm birth and low birth weight [4]. The prevalence of severe obstetric complications, including spontaneous abortion, intrauterine growth retardation, fetal death and premature delivery in Q fever infected women were 26 %, 5.3 %, 5.3 % and 44.7 %, respectively [13]. According to studies, although acute Q fever can be asymptomatic, it can also be associated with severe complications. [14]. Q fever infection in pregnant women and its complications have been neglected in the world and little attention has been paid to it by health systems. Therefore, this systematic review aimed to collect and summarize evidence on the prevalence of Q fever in women and its associated risk factors.

## 2 Methods

### 2.1 Information source and search

This systematic review and meta-analysis was conducted in accordance with the PRISMA (Preferred Reporting Items for Systematic Reviews and Meta-Analyses) guidelines. We searched English electronic data sources including PubMed and Web of science to finding the literature on prevalence of *C. burnetii* in women with a history of abortion in the world, from 1993 to December 2024. In addition, the citations of the included articles were reviewed to find other relevant studies. The keywords that we used for our search were (Q fever OR *Coxiella burnetii*) AND (pregnant women OR miscarriage OR abortion OR aborted).

### 2.2 Eligibility criteria and study selection

The articles that examined Q fever in pregnant women or women with history of abortion in different parts of the world; both by molecular or serological methods were eligible to enter meta-analysis.

Exclusion criteria for this meta-analysis were as follow: 1: Case report articles, 2: Systematics review articles, 3: Lack of access to the full text if there wasn't enough information in the abstract. We also tried to contact the relevant author of the article in case of any problems with the published data or doubts about the eligibility of the articles.

### 2.3 Validity assessment

Four criteria were selected from the Strengthening the Reporting of Observational Studies in Epidemiology (STROBE) to determine the reporting quality of this study: 1. described locations, settings, and relevant dates of study; 2. described the eligibility criteria, 3. clearly define all outcomes, 4. report of the number of interesting outcomes [15]. The studies that meet mentioned criteria were classified as high quality, and studies that did not meet two criteria were classified as low quality.

### 2.4 Data collection and data items

Data were screened by two researchers (ML and SE) and data were extracted for type of study, year and location of study, test method, high-risk groups (contact to animal or living in rural area), and sample size, and number of samples with positive result for *C. burnetii*. In the serological evaluations, both phase I and phase II IgG and IgM antibodies against *C. burnetii* were considered, depending on the diagnostic protocol used in each included study.

### 2.5 Analytic approach

We conducted the meta-analyses in STATA version 17. Meta-analysis of Q fever prevalence in pregnant women based on the method of detection and level of risk group was done. The outcome was measured and reported as pooled prevalence and odds ratio with 95 % confidence intervals. The Q statistic was reported with Chi-square and P values and the I^2^ statistic was reported as a percentage. Der-Simonian–Laird was used as a method for the random effect model. A fixed model was used when the P-Value of the heterogeneity test was>0.1. Meta-regression was used for assessing the heterogeneity reasons for seroprevalence (based on country income situation and year of study).

## 3 Results

### 3.1 Search results

**Fig. 1:**
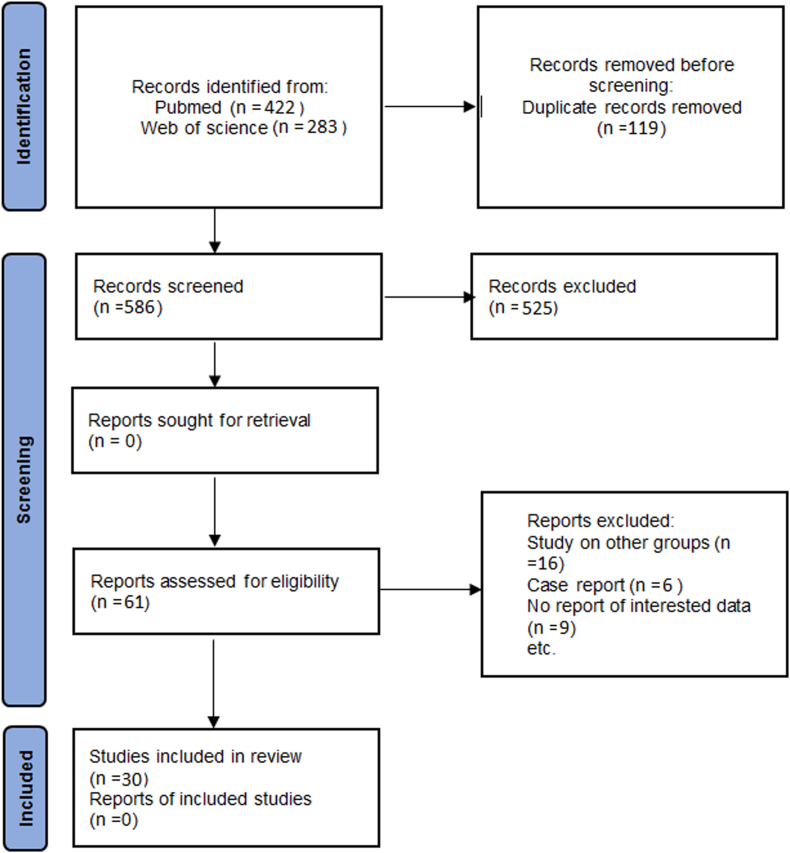
Flow diagram of the systematic review.

In this systematic review, we found 705 records, after removing duplications (n = 119), 586 articles were screened based title and abstract and 525 of them were excluded. The full texts of 61 articles were reviewed, of which 31 were excluded for not meeting the eligibility criteria. Ultimately, 30 studies were included in the meta-analysis (Fig. 1).

1. Prevalence of *Coxiella burnetii* in pregnant or aborted women without infection history
  1.1 Serological result

**Fig. 2:**
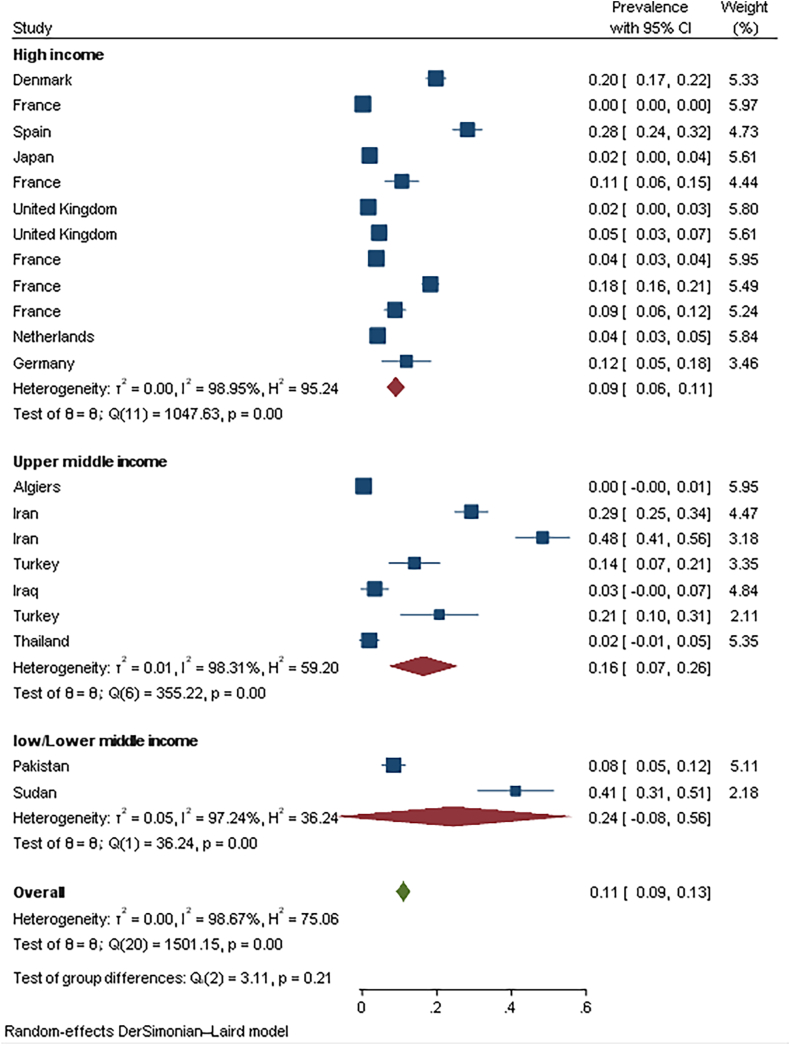
The seroprevalence of Q fever based on the subgroup of countries' income situation.

Totally 21 studies assessed the prevalence of *C. burnetii* via serological assay from 14 different countries (Table 1). The total pooled seroprevalence of *C. burnetii* was 11 % (95 % CI: 9.2–12.9). Among the seropositive group, pooled seroprevalence of IgG antibody was 10.2 %, while pooled seroprevalence of IgM antibody was 1.5 %, indicating recent or acute infection (Table 2, Part A).

1.2 Seroprevalence of Q fever was statistically different in subgroups of countries based on income categories (P = 0.02), and seroprevalence in low/lower-middle income countries (24.4 %, 9 % CI: 0.0–56.4) was higher than in upper-middle-income (16.4 %, 95 % CI: 7.1–25.7) and high-income (9.0 %, 95 % CI: 6.5–11.5) (Fig. 2). Subgroup analysis showed there is significant difference (p = 0.005) between high-risk exposure women (11.2 %) (According to the living area, occupation, and contact with domestic animals) and low risk exposure women (5.1 %). There were no significant difference (p = 0.60) between women with (10.8 %) and without (10.7 %) abortion history. **Molecular result**

**Table 1:** Characteristics of the included studies in the meta-analysis to study the evidence of *Coxiella burnetii* infection among pregnant and aborted women.

	Group category	Country	Year	Method	Sample size	Sample type	Reference
1	Seroprevalence in pregnant women	Algeria	2014–2015	IFA	725	Blood	[16]
2		Iran	2016–2017	ELISA	184	Blood	[17]
3		England	2008	IFA	438	Blood	[18]
4		Iran	2014	ELISA	400	Blood	[19]
5		Netherlands	2007–2008	IFA	1174	Blood	[20]
6		France	2002–2003	IFA	376	Blood	[14]
7		Turkey	2012	IFA	100	Blood	[21]
8		Japan	1993–1998	IFA	200	Blood	[22]
9		France	2013	IFA	179	Blood	[23]
10		Denmark	1996–2003	IFA	856	Blood	[24]
11		France	1996	IFA	12716	Blood	[25]
12		Spain	2009–2010	IFA	500	Blood	[26]
13		France	1997–1998	IFA	7658	Cord blood	[27]
14		France	2014	IFA	1112	Blood	[28]
15		Turkey	2009–2010	IFA	58	Blood	[29]
16		Germany	2003 & 2005	IFA	93	Blood	[30]
17		Thailand	2015–2016	ELISA	105	Blood	[31]
18		United Kingdom	2013–2019	ELISA	369	Serum	[32]
19		Pakistan		ELISA	297	Blood	[33]
20		Sudan	2018	ELISA	90	Blood	[34]
21		Iraq	2022	ELISA	90	Blood	[35]
22	Molecular prevalence in pregnant women	Algeria	2014–2015	PCR	725	Placenta	[16]
23		Turkey	2012	PCR	51	Placenta	[29]
24		France	1997–1998	PCR	246	Placenta	[27]
25		Iraq	2014	PCR	47	Blood	[36]
26		Iran	2021–2022	PCR	409	Placenta and cotyledon	[37]
28	Isolation	Germany	2003 & 2005	Culture	5	Placenta, Amniotic fluid & colostrum	[30]
29	Follow-up of pregnant women with Q fever	France	2006–2011	IFA & PCR	30	Blood & Placenta	[38]
30		India	2007	IFA, PCR & Culture	74	Blood, Genital swabs, Fecal swabs & Urine	[39]
31		Denmark	2007–2001	IFA & PCR	12	Urine, placenta, Bonemarrow, Milk & Blood	[40]
32		Netherlands	2011	IFA & PCR	9	Blood & Placenta	[12]
33		France	2007–2012	PCR & Culture	14	Placenta	[41]
34		France	1997	PCR & Culture	5	Blood, placenta & Fetal Tissue	[11]
35		France	1991–2005	PCR & Culture	53	Placenta & Fetal Tissue	[13]

**Table 2:** The pooled estimate of *C. burnetii* prevalence based on subgroup and method of detection.

Study population	Subgroup	Number of studies	Sample size	Pooled estimate (95 %CI)	I^2^
**Part A: Prevalence of *C. brunetti* in pregnant women without defined previous infection**					
**Serology test**					
Total seroprevalence	Total	21	27720	11.0 (9.2–12.9)	98.68
	IgM	6	3462	1.5 (0.4–2.6)	90.55
	IgG	21	27720	10.2 (8.5–12.0)	98.59
**Seroprevalence based subgroup**					
Income situation	High income	12	25671	9.0 (6.5–11.5)	98.95
	Upper middle income	7	1662	16.4 (7.1–25.7)	98.31
	Low/Lower middle income	2	387	24.4 (0.0–56.4)	97.24
High risk exposure	Yes	12	12942	11.2 (8.4–14.0)	97.97
	No	12	9069	5.1 (3.5–6.7)	96.84
History of abortion	Yes	18	11871	10.8 (8.6–13.1)	97.51
	No	15	11323	10.7 (7.9–13.6)	97.82
**Molecular test**					
**Total prevalence**	**Total**	5	1478	0.1 (0.0–0.4)	68.33
High risk exposure	Yes	2	853	0.3 (0.0–0.7)	57.76
	No	2	281	0.0 (0.0–0.7)	0.38
History of abortion	Yes	4	968	0.6 (0.0–2.0)	76.80
	No	2	264	0.0 (0.0–0.7)	0.00
**Part B: Prevalence of *C. burnetii* in women with the previous infection**					
**Total prevalence**	Total	7	197	62.6 (34.6–90.5)	99.04
**Seroprevalence**	Total	5	55	35.6 (10.0–61.1)	80.15
	IgM	5	178	58.5 (88.1–100.0)	98.09
	IgG	5	178	79.2 (59.4–100.0)	95.35
**Molecular test**	Total	7	145	14.0 (2.7–25.2)	74.56
**Culture**	Total	5	178	79.2 (58.4–100.0)	98.09
High risk exposure	Yes	4	36	72.2 (46.8–97.6)	98.03
	No	4	161	43.3 (0.0–100.0)	98.55
History of abortion	Yes	5	104	62.0 (22.7–100.0)	98.89
	No	5	93	77.6 (39.4–100.0)	96.37

Based on the data of five studies on 1478 women, *C. burnetii* was detected in 0.1 % (95 % CI: 0.0–0.4) of women. The prevalence in high risk exposure women and women with abortion history were 0.3 % (95 % CI: 0.0–0.7) and 0.6 % (95 % CI: 0.0–2.0) respectively (Table 2, Part A).

2. Prevalence of *Coxiella burnetii* in pregnant or aborted women with an infection history

**Fig. 3:**
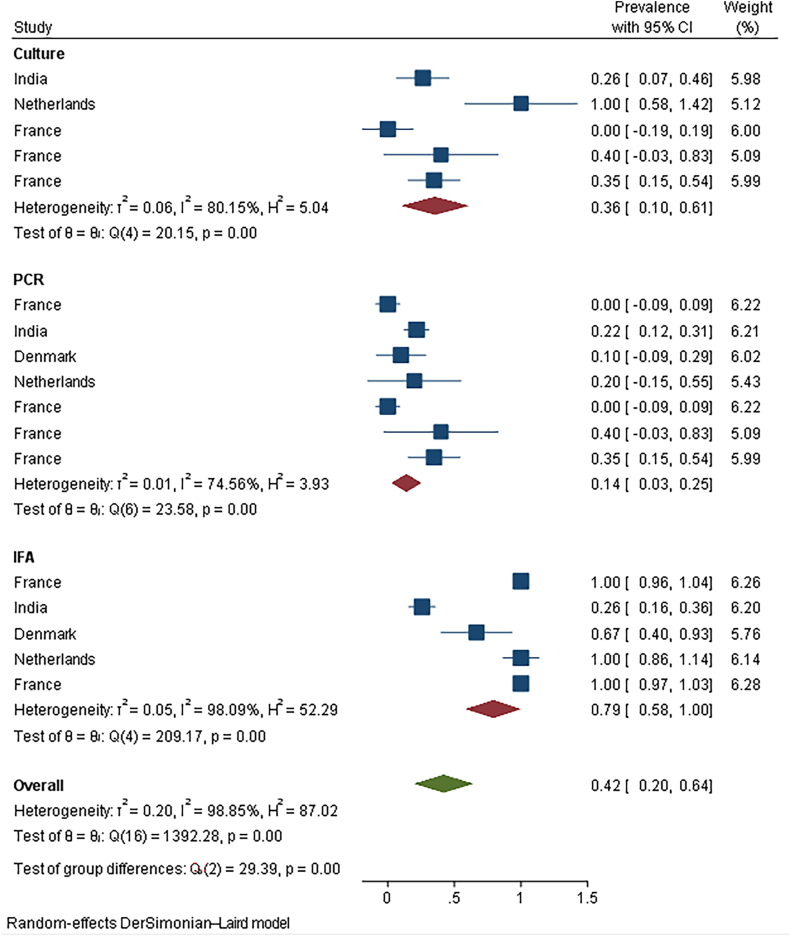
The prevalence C. burnetii in women with previous infection history in follow-up duration based on the method of detection.

Total prevalence in women with previous infection was 62.6 % (95 %CI: 34.6–90.5).In this group pooled seroprevalence of IgG antibody was 79.2 %, while pooled seroprevalence of IgM antibody was 58.5 %. Prevalence of *C. burnetii* in women with and without high risk exposure were 72.2 % and 43.3 % (Fig. 3). Results showed 62.0 % and 77.6 % of women with abortion and without abortion were positive for (Table 2, part B).

## 4 Discussion

Abortion is a serious complication in pregnant women, occurring in 10–15 % of cases, especially during the first trimester. Infection with intracellular bacteria such as *Listeria* and *Coxiella burnetii* can lead to adverse outcomes, including infertility, stillbirth, preterm delivery, ectopic pregnancy, intrauterine fetal demise, and low birth weight. These bacteria are food-borne and zoonotic pathogens that target embryonic tissues and can cause fatal diseases in both the mother and fetus [42,43].The result of our study showed that the pooled seroprevalence of Q fever among pregnant women was 11 %. Also, in seroprevalence studies, most positive cases belonged to the high-risk group (11.2 %), but a history of abortion/without abortion (10.8 %/10.7 %) had no effect on the results. According to the molecular method the prevalence of Q fever was 0.1 % and all the of the positive result belonged to the women with history of abortion (0.6 %) and high risk exposure (0.3 %).

In general, the pathogenesis of the disease in pregnant women has not been fully identified, but some studies have isolated the bacterium from the placenta, such as a study in Algeria that collected 380 placental samples from women who had a spontaneous abortion and 4 cases were reported positive by molecular method [16].

Subgroup analysis in our study did not show significant differences between women with and without a history of abortion. Similarly, some studies have not found evidence of a higher prevalence of Q fever infection among individuals with a history of abortion. Determining the true impact of Q fever infection on pregnancy outcomes regardless of abortion history is challenging based on the available data [44]. Additionally, since most cases of Q fever are asymptomatic, underreporting in studies may have influenced the results. In this study, we found a significant relationship between women with high risk exposure (11.2 %) and the seroprevalence of *C. burnetii*.

According to the study in endemic areas of France for Q fever, 7658 cord blood samples were examined to identify antibodies against *C. burnetii* by IFA, and 3.8 % of sample positive for Q fever, which was significantly associated with fetal death [27]. Studies examining the serum prevalence of Q fever in pregnant women show variable prevalence in endemic areas: 2.6 % in France [45], 3.8 % in Canada [46], 9.1 % in the Netherlands [47], and 4.6 % in the United Kingdom [48]. Living in high-risk areas, as well as having close contact with domestic animals and livestock, increases the risk of infection in pregnant women. May be living within 5 km of farms with infected goats and sheep is considered a high-risk area for the disease. Also, travel to these areas can cause infection in pregnant women [49]. In Denmark, the seroprevalence of *C. burnetii* was 47 % in pregnant women who had occupational exposure to livestock, while in women who had no exposure to livestock, the prevalence was 4.8 % [24]. According to the results of another study in Denmark on 397 blood samples of pregnant women who were exposed to cattle or sheep at home or occupation, 147 (37 %) of samples were reported positive by serological methods and had antibodies against *C. burnetii* [24].

In situations where avoiding contact with livestock is not feasible, additional preventive measures can reduce the risk of *C. burnetii* infection. These include the use of protective equipment such as masks and gloves in endemic region. Maintaining appropriate hand hygiene and applying disinfectants such as 10 % household bleach or 70 % ethanol on contaminated surfaces are also effective in reducing environmental transmission. Furthermore, minimizing exposure to ticks, which can act as vectors, is advised through the use of repellents and protective clothing. Although a vaccine against Q fever (Q-Vax®) is currently available and approved only in Australia, its use is limited to high-risk occupational groups and not globally implemented [50]. Therefore, non-vaccine preventive strategies remain critical in most regions.

Based on the analysis of our results, most seropositive cases of Q fever were associated with low-income areas (24.4 %) compared to middle-income (16.4 %) and high-income (9.0 %) areas. Similarly, a study conducted on pregnant women in Pakistan reported a significantly higher Q fever seroprevalence in women from low-income areas (13.7 %) compared to those from medium- and high-income areas (3.6 % and 6.2 %, respectively). This may be attributed to the zoonotic nature of the disease and the increased likelihood of contact with livestock or agricultural environments among women living in low-income regions [33]. According to the One Health theory, emerging zoonoses particularly in endemic areas have a greater social impact on low-income populations, necessitating increased attention and public health intervention [51].

Our study also found that the prevalence of Q fever among pregnant women with a previous history of infection was 62.6 %, with most cases associated with residence in high-risk areas and a history of miscarriage (72.2 % and 62 %, respectively). *C. burnetii* infection is considered serious not only during the pregnancy in which it occurs but also in subsequent pregnancies if left untreated [52]. Additionally, a study conducted in the Netherlands, in areas affected by two major Q fever outbreaks between 2007 and 2009, and examined 2004 pregnant women for antibodies against *C. burnetii*. Using the immunofluorescence method to detect phase I and phase II antibodies, 181 samples were found positive for IgG phase II antibodies [53]. Another study in France reported that two-thirds of pregnant women with untreated Q fever were at risk of abortion, while one-third faced a risk of premature birth. These findings underscore the importance of monitoring and treating Q fever during pregnancy to prevent complications in both current and future pregnancies [54].To date, no formal global study has specifically examined the geographical distribution of Q fever in pregnant women. However, according to a report by the World Health Organization, Q fever has been documented in 51 countries across five continents, indicating its widespread presence [55]. In our review, the majority of included studies originated from France, which also contributed the largest study populations. This concentration of data from Europe and the Middle East limits the generalizability of our findings to other regions. Given the potential severity of Q fever during pregnancy, greater attention and surveillance efforts are warranted in endemic areas where livestock exposure is common. Future research should aim to address this gap and better characterize the burden of Q fever among pregnant women globally.

Several methods have been proposed to identify *C. burnetii* in clinical specimens. These methods include molecular methods, serological methods (ELISA & IFA) and culture, which can be used to more quickly identify the disease and prevent its dangerous side effects [39]. In general, treating Q fever during pregnancy is associated with great challenges. Since doxycycline is a choice drug for the treatment of Q fever and this drug is contraindicated during pregnancy, as a result, the course of treatment with cotrimoxazole has been suggested since 1996 [56]. In a prospective study conducted in France, a significant association was observed between Q fever and adverse pregnancy outcomes, including miscarriage, preterm delivery, and stillbirth. In this study, 19 out of 179 pregnant women (10.6 %) with unexplained pregnancy complications tested serologically positive for *Coxiella burnetii*. The cumulative incidence of Q fever-related pregnancy complications in the reproductive population was estimated to range between 2.2 and 5.2 per 1,000, a rate comparable to that of TORCH pathogens (toxoplasmosis, others (syphilis, hepatitis B), rubella, cytomegalovirus, herpes simplex) in the same region. These findings highlight the considerable burden of Q fever in endemic areas with high levels of livestock exposure. Therefore, screening at-risk women and minimizing contact with livestock during pregnancy may help reduce the impact of the disease [57].

In France, Q fever is a notifiable disease, and *C. burnetii* testing may be conducted in pregnant women with relevant clinical symptoms or exposure history, particularly in endemic regions [58]. Following a large outbreak in the Netherlands (2007–2010), heightened awareness led to increased testing and surveillance, but routine screening of pregnant women was not adopted nationally [59]. However, to date, there is no international guideline recommending routine Q fever screening during early pregnancy or after spontaneous abortion, and its implementation remains limited to specific clinical indications or outbreak settings.

## 5 Conclusions

According to the results of studies and the risk of infection of pregnant women with Q fever and obstetric complications of this disease, the health system should more pay attention to Q fever, especially in endemic areas of this disease. Given the limited information about the pathogenesis of Q fever during pregnancy, serological screening of women with occupational exposure to livestock or residing in endemic areas may be advisable, particularly in the context of targeted public health interventions It is also necessary to follow up on the treatment of pregnant women who have a history of Q fever infection to control the disease and prevent chronic Q fever infection, as well as complications in subsequent pregnancies.

## CRediT authorship contribution statement

**Mina Latifian:** Writing – original draft, Investigation, Data curation. **Fahimeh Bagheri Amiri:** Writing – review & editing, Formal analysis, Data curation. **Ehsan Mostafavi:** Writing – review & editing, Data curation. **Saber Esmaeili:** Writing – original draft, Project administration, Methodology, Data curation, Conceptualization.

## Ethics approval and consent to participate

Not applicable.

## Consent for publication

Not applicable.

## Availability of data and materials

No data was used for the research described in the article.

## Fundings

Not applicable.

## Declaration of competing interest

The authors declare that they have no known competing financial interests or personal relationships that could have appeared to influence the work reported in this paper.
